# Achieving decent living standards in emerging economies challenges national mitigation goals for CO_2_ emissions

**DOI:** 10.1038/s41467-023-42079-8

**Published:** 2023-10-10

**Authors:** Jingwen Huo, Jing Meng, Heran Zheng, Priti Parikh, Dabo Guan

**Affiliations:** 1 Department of Earth System Science, Ministry of Education Key Laboratory for Earth System Modeling, Institute for Global Change Studies, Tsinghua University, Beijing, 100084 China; 2 The Bartlett School of Sustainable Construction, University College London, London, WC1E 6BT UK

**Keywords:** Climate-change mitigation, Climate-change policy, Energy and society

## Abstract

Emerging economies, low- and middle-income countries experiencing rapid population and GDP growth, face the challenge of improving their living standards while stabilizing CO_2_ emissions to meet net-zero goals. In this study, we quantify the CO_2_ emissions required for achieving decent living standards (DLS) in emerging economies. The results show that, compared to other regions, achieving DLS in emerging Asian and African economies will result in more additional CO_2_ emissions, particularly in the DLS indicators of Mobility and Electricity. Achievement of DLS in emerging economies will result in 8.6 Gt of additional CO_2_ emissions, which should not jeopardize global climate targets. However, a concerning trend arises as more than half of the emerging economies (62 out of 121) will face substantial challenges in aligning their expected emission growth for achieving DLS with their national emission mitigation targets.

## Abstract (web summary)

Achieving decent living standards for global emerging economies is estimated to lead to an additional 8.6 Gt of CO_2_ emission with more than half of emerging economies emitting additional CO_2_ more than the value of their emission reduction commitments

## Introduction

In order to address the negative impacts of climate change, the Intergovernmental Panel on Climate Change (IPCC) of the United Nations has proposed that the world must halve net anthropogenic CO_2_ emissions within a decade^1^ and achieve carbon neutrality by 2050^2,3^. Emerging economies, representing low- and middle-income countries with rapid population growth and comprising more than 80% of the global population^4,5^, are playing an increasingly important role in global GDP growth^6–8^. However, emerging economies are facing multiple challenges, including the need to rapidly improve living standards for human development in urbanization and population growth^9,10^. A large proportion of the population in emerging economies currently has low levels of resource consumption and hence emissions due to lower living standards. Essential energy is required to meet the basic living needs of everyone^11^. However, emerging economies will experience higher energy demands, particularly for fossil fuels, as they undergo industrialization and have relatively outdated energy infrastructures. Consequently, an improvement in living standards within these emerging economies may result in a subsequent increase in CO_2_ emissions associated with fossil fuels in the future^12,13^.

Decent living standards (DLS) is a key assessment framework comprising multidimensional indicators at household, community, and national levels^14,15^, which reflects the basic material requirements to achieve human prosperity and well-being^16^. Achieving a decent life as soon as possible is of great significance for achieving the SDG goals (Sustainable Development Goals), protecting the environment, and improving human well-being^14^. Some researchers have quantified the energy demand for achieving DLS globally^17^ and explored the potential trade-offs between DLS achievement and emission mitigation^15^. Previous studies have also calculated the energy demand for achieving DLS in several specific emerging economies^18^. Given the large number of emerging economies and the likely contribution to emissions they will make in the future, it is vital to investigate the potential implications of DLS achievement in the current emission-constrained world. However, the extent of additional CO_2_ emissions that would be generated to achieve a decent life and the implications on national carbon emission targets, especially for all emerging economies, is still unclear.

Here, we use the full-scale, near real-time multi-regional input-output model for the global set of emerging economies (EMERGING MRIO)^19^, which covers global 245 economies, 135 economic sectors (105 commodity sectors, and 30 service sectors) and is the latest updated to 2019. We aim to assess the additional CO_2_ emissions that would arise in all emerging economies globally (Supplementary Fig. 2) to achieve DLS and LS at different development stages. Furthermore, we analyze the regional heterogeneity of emissions budgets for 10 DLS indicators and explore the potential impact of achieving DLS on the emission reduction commitments of emerging economies in the future

Building on the definition of DLS^14^, we select 10 indicators related to expenditure for quantification: Food, Clothing, Housing, Sanitation, Health, Education, Water, Electricity, Mobility, and ICT (access to phones, TV, and internet services). In this paper, the scope of CO_2_ emissions only includes CO_2_ generated by fossil fuel combustion.

## Results

### Heterogeneity between countries in DLS indicators in 2019

**Fig. 1:**
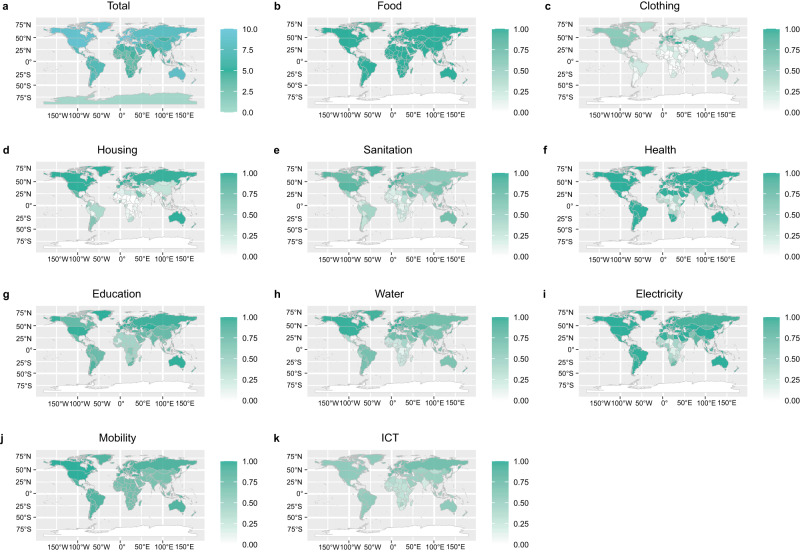
The decent living standards (DLS) indicator map for 245 countries and regions in 2019. **a** The total DLS value ranges from 0 to 10. **b**–**k** The value of 10 DLS dimensions in Food, Clothing, Housing, Sanitation, Health, Education, Water, Electricity, Mobility, and ICT, ranges from 0 to 1. The base map is from the mapdata package (TM World Borders Dataset 0.3) in R (https://search.r-project.org/CRAN/refmans/prevR/html/TMWorldBorders.html).

The DLS indicators range from 0 to 1, representing the percentage of the population in each country for whom the standard of living has reached DLS. We assume that 10 DLS indicators have the same contribution to the total DLS of the country, so the total value of national DLS is the sum of the 10 DLS indicators, ranging from 0 to 10. We observe a considerable difference in the value of the DLS indicators between different regions, especially between developed countries and emerging economies (Fig. 1, Supplementary Data 4). In general, the overall difference in the total value of the DLS indicators between different regions is mainly influenced by the regional level of development.

Western Europe and North America, which mainly consist of developed countries, have relatively narrow gaps in the DLS indicators. Specifically, all populations in these regions meet the DLS thresholds for Food and Health, while over 80% of the population in 12 out of 19 countries meet DLS thresholds for Education, Water, Sanitation, Housing, and Mobility. However, almost 40% of the population in 8 out of the 19 countries in these regions lacks access to ICT, such as in the USA and Canada. In contrast, the gaps in the DLS indicators are wider in regions with more emerging economies. Due to its relatively low level of overall development, Africa exhibits lower average living standards than other regions of the world, and the average estimated total value of DLS is only 4.3. In 47 out of 58 African countries, more than 50% of the population does not meet the DLS thresholds for Education, Water, Sanitation, and Clothing. In Nigeria, for example, more than 65% of the population does not meet the DLS minimum standard in more than half of the DLS indicators, primarily in Clothing, Housing, Sanitation, Health, Water, and ICT. In Latin America, Oceania, and Asia, low capacity to deliver basic materials and social services results in large gaps in Sanitation, Water, Clothing, ICT, and Housing. For example, in 42 out of 56 Latin American countries, more than 40% of the population does not meet the DLS thresholds for ICT and Sanitation. In 8 out of 27 countries of Oceania, more than 50% of the population lives below the DLS thresholds. Similarly, in 37 out of 53 Asian countries, 40% and 30% of the population do not meet the DLS thresholds of ICT and Sanitation, respectively. For example, Brazil’s ICT and Sanitation indicators are 0.59 and 0.49, respectively. In addition, 30% of the population in China does not have access to safe sanitation.

The gap between different DLS indicators is not only related to regional levels of development but is also closely related to regional climatic conditions and access to infrastructure. For example, the Food indicator performs well in most countries. However, the Clothing and Housing indicators for each country are not only related to the level of development and consumption but are also influenced by the regional climate zone. For example, the rate of demand for air conditioning (AC) is an essential factor in the housing index. Southeast Asian countries in the subtropical region have a higher demand for AC than European countries in the higher latitudes of the northern hemisphere. Due to their low levels of infrastructure development, Africa and Asia face critical gaps in DLS indicators, including Water, Education, Sanitation, Health, Electricity, Mobility, and ICT. For example, in over 93% of African countries, more than half of the population does not have access to safe drinking water. Ethiopia has an ICT indicator of 0.32, while Uganda has a Health indicator of 0.21. In India, the Water and Sanitation indicators are 0.7 and 0.36, respectively.

### Emissions of achieving decent living in emerging economies

**Fig. 2:**
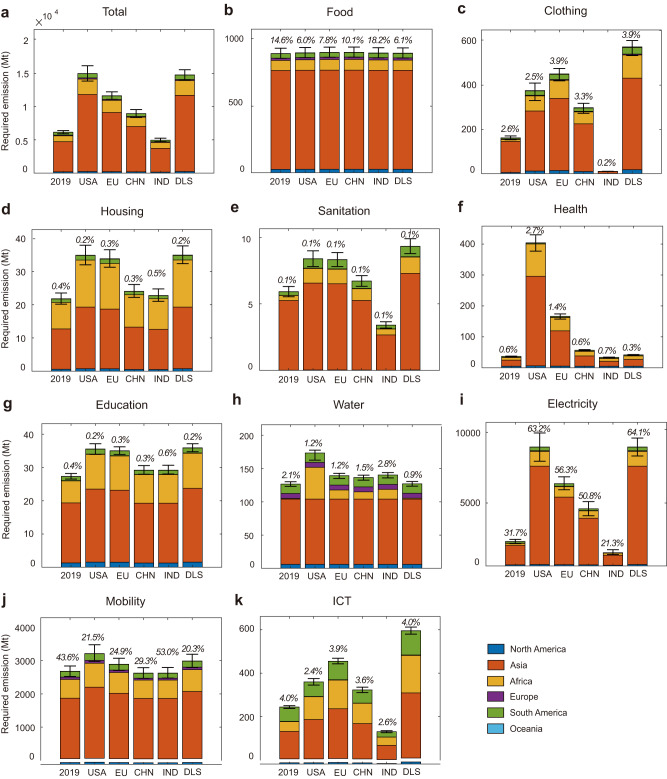
The generated emissions for achieving decent living standards in emerging economies in 2019. **a** The total generated emissions for achieving DLS. **b**–**k** The generated emissions for achieving DLS by ten indicators, i.e., Food, Clothing, Housing, Sanitation, Health, Education, Water, Electricity, Mobility, and ICT. Bars show the generated emissions for achieving five different LS baselines: “2019”: CO_2_ emissions generated to meet the current LS for 10 DLS indicators in 2019; “USA”: meets the LS of USA; “EU”: meets the mean LS of EU27 + UK; “CHN”: meets the LS of China; “IND”: meets the LS of India; “DLS”: meets the DLS, which means all DLS indicators of emerging economies are 1. Colors present different regions. The numbers above the column bar present the proportion of different DLS indicators to the total emissions in each scenario. Under the same LS scenario, the sum of the number on the columns in the (**b**–**k**) is 100%. The error bar represents a 95% confidence interval (CI), determined by the uncertainty of DLS indicator data and carbon emission intensity.

We design five LS achievement counterfactual scenarios to quantify the impact of achieving DLS and LS at different development stages: United States (LS-USA), mean LS of the European Union and the United Kingdom (EU27 + UK), China (LS-CHN) and India (LS-IND). We assume that global emerging economies will achieve different LS as soon as possible, while keeping their emissions intensity and production structure unchanged from 2019. If the global emerging economies achieve decent living (“DLS” line), the CO_2_ emissions resulting from increased consumption will reach 14.7 Gt, which is 8.6 Gt more than the emissions for LS in 2019 (Fig. 2a, Supplementary Fig. 2). The main factors contributing to additional CO_2_ emissions for DLS are Electricity and Mobility. Due to differences in consumption demand and carbon intensity of emerging economies, the contribution of six regions to additional CO_2_ emissions for different indicators is heterogeneous. Due to its large population and high carbon emission intensity, Asia is the main contributor of additional CO_2_ emissions generated to achieve DLS for Food, Clothing, Sanitation, Water, Health, Electricity, Education, and Mobility. For example, the share of additional CO_2_ emissions generated for Electricity and Mobility in Asia is up to 82.7% and 67.4%, respectively. In terms of ICT, South America and Africa contribute the most to the additionally generated CO_2_ emissions, accounting for up to 47.7%. Due to their lower levels of development, Asia and Africa also contribute more to the generated emissions in the Housing indicator, with more than 53.1% and 40.5%, respectively.

The impact of achieving different LS on the additional CO_2_ emissions generated by emerging economies varies among different DLS indicators. In general, the United States has a higher living standard than the EU27 + UK, which requires emerging economies (especially in Asia and Africa) to emit an additional 3.3 Gt of CO_2_. On the other hand, living standards in India and China are relatively low for emerging economies. India’s LS is even lower than the average level for global emerging economies. Hence, emerging economies can reach the same living standards as China with only 2.8 Gt of additional emissions and do not generate any additional emissions overall to achieve the same living standards as India in 2019, although living standards in India are likely to rise over the next decade. It’s important to note that while China and India have lower per capita living standards, they are populous nations with nearly 2.8 billion people. They, therefore, need to prioritize low-carbon transformations while pursuing the DLS (Fig. 2a). The United States has higher living standards for Food, Health, Water, and Mobility than the minimum thresholds set by the DLS. For example, healthcare expenditure per capita in the US is 10.9 thousand USD in PPP term, and 99% of the population has access to safe water supply with a withdrawal rate of 3.7t/cap/day. Moreover, the car and public transport occupancy rate is 93% of total mobility. Therefore, to reach the LS-USA, 590.1 Mt, 967.7 Mt, 992.6 Mt, and 633.9 Mt of additional CO_2_ emissions are generated for Food, Health, Water, and Mobility respectively, compared to the LS of EU27 + UK, LS-CHN, LS-IND and DLS.

On the other hand, the mean LS of the EU27 + UK for Clothing and ICT is much higher than the LS-USA. People in the EU27 + UK spend PPP$394 per capita on clothing, compared to PPP$326 per capita in the USA. Additionally, 76.4% of the population in the EU27 + UK have access to phones, TV, and the internet, compared to 60% in the USA. Therefore, reaching the LS of the EU27 + UK results in an additional 75.5 Mt and 95.0 Mt of CO_2_ emissions for these two DLS indicators compared to the LS-USA. As emerging economies, China and India have relatively lower living standards than the global average LS of emerging economies in terms of Sanitation, Housing, Clothing, and Electricity. In India, for example, only 36.5% of the population has access to safe sanitation (Fig. 2e). Thus, emerging economies will save 2.5 Mt of CO_2_ emissions for the Sanitation indicator in the LS-IND compared to emissions in 2019 (5.9 Mt), even though India’s access to sanitation services will increase in the future. To achieve 100% population coverage in meeting the DLS for ICT, Clothing, and Sanitation, emerging economies will need an additional 351.2 Mt, 411.4 Mt, and 3.4 Mt of CO_2_ emissions, respectively, compared with the CO_2_ emissions in 2019.

### Impact of achieving decent living on national emission reductions

Setting emission reduction targets is crucial to achieving the goals of the Paris Agreement, which outlines the GHG emission reductions that countries have committed to achieve by 2030 or 2050^20,21^. To this end, over 158 countries, collectively responsible for around 88% of global GHG emissions in 2019, have developed policies and strategies to reduce emissions and achieve carbon neutrality. In this study, we use national emission mitigation data for 121 emerging economies from the Net Zero Tracker^22^, which gathers policy information on net zero emission targets set by countries. Our results focus exclusively on CO_2_ emission mitigation as part of GHG emissions.

**Fig. 3:**
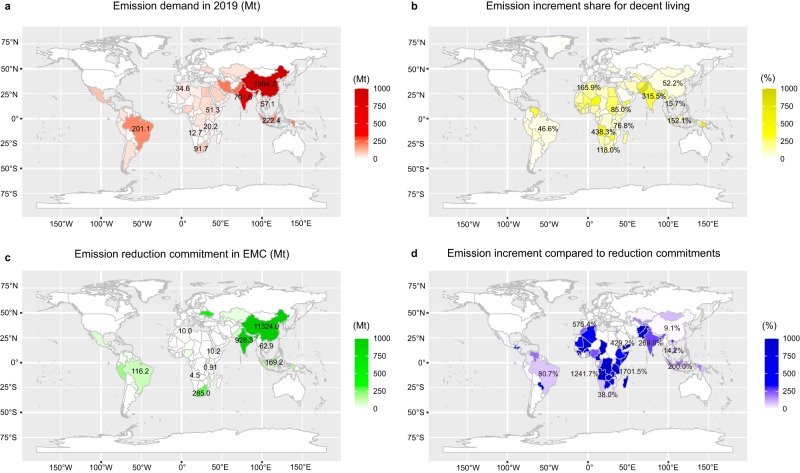
The generated CO_2_ emissions for achieving decent living and emissions mitigation for national emission reduction commitments in emerging economies. **a** The CO_2_ emissions for LS in 2019; **b** incremental shares of emissions for achieving DLS compared with CO_2_ emissions in 2019; **c** CO_2_ emission reduction commitments^17,18^, and **d** the expected CO_2_ emission increment compared to reduction commitments: CO_2_ emission increment/CO_2_ emission reduction commitment. “EMC” means National emission reduction commitments. The base map is from the mapdata package (TM World Borders Dataset 0.3) in R (https://search.r-project.org/CRAN/refmans/prevR/html/TMWorldBorders.html).

Achieving DLS in emerging economies may necessitate additional CO_2_ emissions, thereby placing a heightened burden on emission reduction efforts and potentially impeding their ability to meet emission reduction targets. Figure 3 shows that 121 emerging economies worldwide have issued policies with varying degrees of emission reduction effort, with 62 economies (51.2%) expected to emit more to reach DLS than the CO_2_ emissions value in their emission reduction commitments. India, for example, may result in an increase of CO_2_ emissions by 315.5% (2.5 Gt) to achieve DLS compared to 2019. This is despite India’s commitment to reducing CO_2_ emissions by 928.3 Mt, which is equivalent to 40.4% of India’s total production-based CO_2_ emissions in 2019 (2.3 Gt). Additionally, 30 out of 45 emerging economies in Africa may face a threefold increase in CO_2_ emissions for achieving DLS, making it more challenging to achieve national carbon emission reduction policies without international assistance. For example, Algeria has committed to reducing CO_2_ emissions by 10.0 Mt in the future, but an increase in emissions of 57.3 Mt is generated to reach DLS, representing 40.2% of total national production-based CO_2_ emissions in 2019 (142.4 Mt). Algeria’s CO_2_ emission increment is 5.8 times that of its emission reduction commitment. Kenya, on the other hand, has committed to reducing CO_2_ emissions by 6.4 Mt, while an emission increase of 25.9 Mt is generated to achieve the DLS, which is 128.9% of the national total production-based CO_2_ emissions in 2019 (20.1 Mt) and is 4.0 times that of Kenya’s emission reduction commitment. (see more details in Supplementary Data 5 and Supplementary Fig. 1).

## Discussion

This study provides an analysis of the additional CO_2_ emissions generated to achieve DLS in emerging economies. Our results show that rapidly achieving DLS in emerging economies need only generate 8.6 Gt of additional CO_2_ emissions and has no impact on global climate goals, similar to poverty reduction efforts reported in previous studies^13,23^. Moreover, our study quantifies the challenge that emerging economies face in meeting their carbon reduction commitments while achieving DLS thereby highlighting the need for CO_2_ emissions growth in emerging economies to achieve decent living for all.^15^. The challenge for emerging economies is, therefore, to ensure that they adopt green and sustainable development pathways to improve access to basic services such as infrastructure and reduce CO_2_ emissions.

Increasing investment in green transportation and green power generation is the main pathway to reducing emissions while achieving DLS. Our results indicate that Mobility and Electricity are the main contributors to CO_2_ emissions in emerging economies, particularly in Asia and Africa. Shared infrastructure can help decrease per capita emissions intensity^24^. Governments should prioritize investment and promotion of public transportation to slow the growth of private cars. Investment in active transport infrastructure, like bike lanes and sidewalks, and digital trade and intelligent supply chain management can also reduce transportation needs and thus decrease CO_2_ emissions^1,25^. In addition, promoting the development of new energy vehicles can help lower the carbon intensity of transportation per capita^26^.

In the future, the electricity demand is expected to continue increasing, especially in Asia and Africa. As emerging economies develop rapidly, it is crucial to prioritize green and low-carbon power generation. Renewable energy sources, such as sustainable biofuels, wind power, solar power, and low-emission hydrogen, will play an important role in the power system^1,25^. Studies have shown that the unit cost of solar and wind power is lower than that of any other power source, making them the economical and environmentally friendly choice for new capacity needs in emerging economies^27^. Currently, emerging economies rely heavily on fossil energy power generation. For example, coal power generation has become the main power source in India, accounting for about 70% of the total power generation capacity. This reliance on coal is concerning, particularly when considering the high emission intensity of coal power plants in India, reaching up to 926 g CO_2_e/kWh^28,29^. These figures underscore the imperative for emerging economies to curtail their overall reliance on fossil fuels and seriously consider the implementation of carbon capture and storage (CCS) technologies within their remaining fossil fuel power systems^1^. However, the widespread adoption of CCS is hindered by factors such as cost, energy loss, technical barriers, incumbent supply chains, and the selection of storage sites. The high cost of CCS is identified as the main challenge to its future deployment^30^.

Reducing consumption and waste in developed countries can compensate for CO_2_ emissions generated by emerging economies to achieve DLS^31–33^. Rao et al. ^34^ proposed the concept of a “carbon emission space”, the finite amount of CO_2_ emissions that can be released into the atmosphere without causing further global warming or climate change, suggesting that this space should be allocated fairly among all countries with the necessary energy supply capacity built in to achieve DLS. Our findings indicate that the living standards of developed countries in 2019 exceeded the DLS threshold in terms of Food, Water, Mobility, and Health, which could create a surplus of 572.2 Mt emission space for emerging economies to achieve DLS (Supplementary Table 4). This suggests that consumption levels, and therefore CO_2_ emissions, are very high in developed countries and that there is space for reducing CO_2_ emissions by minimizing unnecessary consumption. Numerous studies have demonstrated that by allocating resources and organizing the economy based on principles of fairness and sufficiency, the world can consume less energy while still achieving high levels of human well-being^17,35^. This approach, in theory, makes it easier to reduce CO_2_ emissions rapidly and improve socio-economic outcomes. Developed countries’ consumption will result in a large amount of extra emissions beyond meeting basic DLS^36^. Therefore, changing the consumption lifestyles of developed countries can be a highly effective mechanism for offsetting additional emissions and conserving emissions budgets for emerging economies to achieve DLS^37,38^. This presents an opportunity to explore trade-offs where developed countries can reduce their CO_2_ emissions to enable development and an improvement in living standards for emerging economies.

Promoting international assistance is necessary for emerging economies to achieve DLS under emission mitigation. Climate justice dictates that “equitable access to basic services and achieving sustainable development for all must be achieved^39^. Emerging economies face challenges in achieving sustainable development and reducing emissions simultaneously due to ongoing industrialization and high carbon intensity^40^. Thus, international assistance will play a crucial role in supporting climate change mitigation and improving the well-being of emerging economies. Accelerating targeted financial support for green technology transfer will address the inequality in access to financial resources for mitigating climate change in emerging economies. Public funding and climate finance for vulnerable areas such as Africa will be cost-effective and offer a high social return^1,25^. Simultaneously, increasing international financial, technological, and capacity-building support and strengthening innovation technology and knowledge transfer will accelerate the global spread of CO_2_ emission reduction technologies, practices, and policies in emerging economies, helping them implement carbon emission reduction policies consistent with other development goals^41,42^. The loss and damage fund agreed at COP27 has been 30 years in the making and will provide opportunities for targeted financial flows into emerging economies to address the impact of climate change. The funds could be directed toward housing, infrastructure, and transportation needs to improve living standards. To ensure the new infrastructure and housing built is climate resilient and low-carbon, green technologies, and production will be crucial. Achieving this requires low-carbon technical innovation and knowledge transfer to emerging economies for their green production^43^.

There are several uncertainties and limitations in our analysis. Firstly, the 10 DLS indicators analyzed in our study are aggregated and may not capture the detailed aspects of the living standards as defined by the DLS framework^14^. Secondly, as we only focused on CO_2_ emissions in this paper, we assumed that the single CO_2_ emission reduction target is consistent with the overall GHG target to calculate the future carbon emission reductions of different emerging economies. Therefore, we quantify these uncertainties and find that the generated CO_2_ emissions in emerging economies for LS in 2019 and for achieving DLS are 6.1 Gt (95% confidence interval of 5.9–6.4 Gt) and 14.8 Gt (95% confidence interval of 14.0–15.5 Gt), respectively (Supplementary Table 5).

In the future, we will further disaggregate these 10 merged indicators into more detailed DLS indicators and plan to collect DLS indicator data on individual and household consumption levels in order to more accurately quantify the increase in CO_2_ emissions resulting from emerging economies to achieve DLS. Specifically, we will calibrate the current global unified minimum threshold of DLS indicators according to the characteristics of individual and household consumer behavior in different countries. We will also consider quantifying the incremental changes in CO_2_ emissions over time, considering changes in carbon emission intensity of DLS indicators, national production structures, and consumption structures. This enables us to conduct a comprehensive analysis of the heterogeneity of different DLS indicators on a more refined scale in order to more effectively formulate sustainable development strategies for different emerging economies.

## Methods

### Data source

We utilized the EMERGING MRIO Database in 2019 with 245 economies and 135 sectors in this research^19^ (Supplementary Data 1 and 2). The historical energy-related CO_2_ emissions data for MRIO are from the International Energy Agency (IEA) database^44^ and CEADs emission inventory data^45^ (for 50 emerging economies; see the country list in Supplementary Table 3). The raw data sources for the 10 DLS indicators are listed in Supplementary Table 1.

### The EMERGING MRIO

Emerging economies are playing an increasingly important role in the global supply chain in the context of globalization. At the same time, these economies face multiple challenges, including population explosion, poverty, and climate change, which can be amplified in the supply chain^9,10^. However, due to difficulties in data collection and the constraints of data compilation, the existing multi-regional input–output (MRIO) databases do not reflect the connection with enough regional and sector details^46,47^, especially for emerging economies, which impedes the analysis of historical supply chains and international trade patterns, and the forecast of future trends.

To fill this gap, we have proposed a modular compilation framework method for MRIO, the EMERGING model. The EMERGING model is a global MRIO framework based on bilateral trade data and national statistics at the individual country level. The contributions are (1) global scale and including emerging economics to the largest extent; (2) containing enough detail on sectors to capture structural changes in supply chains and economic developments; (3) covering changes over time; (4) up-to-date representation of changes to allow for timely policy implications; and (5) using modular compilation for timely updates.

Based on this model framework, the EMERGING MRIO database now covers 135 sectors in 245 economies over the period 2015–2019. It will be an essential tool for conducting supply chain and environmental impact analysis, especially for global emerging economies.

The methodology paper on EMERGING MRIO construction was published in the *Journal of Industrial Ecology*^19^. The full database is open access: CEADs website (https://ceads.net/user/index.php?id=1274&lang=en).

### DLS indicator processing

#### Food

Food requirements are usually characterized by the average calories consumed per day. We determine the calorie gap based on the calorie requirement per capita per day (in kcal) from the FAO^48^. Due to the different calorie requirements of men, women, and children, we derive the national average threshold value of calorie requirements according to the population structure of each country^49^, which is listed in Supplementary Table 2. The intensity at the base year is derived by dividing total embodied emissions in food from the MRIO model by the total calorie intake.

#### ICT (access to phones, TV, and Internet services)

For ICT, we set a normative DLS threshold for all the countries where 100 percent of the households have access to phones, TV, and internet services. The national gap in ICT is based on the data on household access to phones, TV, and Internet services (%) from UN data^50^. Then we apply the emissions intensity of the ICT sector (CO_2_ Mt per dollar) by using EMERGING MRIO to estimate emission requirements in the ICT indicator.

#### Education

For Education, the data on educational attainment, at least completed primary education (population 25+ years %) is from the World Bank^51^. We use a similar approach, considering all countries where 100 percent of the population has completed primary education. In this paper, we track the percentage of adults (aged 25 years and older) who have completed the lowest level of education defined in the International Standard Classification of Education (ISCED), primary education. Then we apply the emissions intensity of the Education sector (CO_2_ Mt per dollar) by using EMERGING MRIO to estimate emission requirements in the Education indicator.

#### Mobility

We use the passenger-kilometer (p-km) as the unit of Mobility indicator, which means that one passenger is transported one kilometer by specific transportation modes (highway, railway, air transport, sea transport, etc.)^34^. Due to affluence, everyone has access to motorized transport, reflecting the lowest levels of average transport demand among industrialized countries. The current share data on transport mode is from ITF Transport Statistics^52^ (Supplementary Data 3). We estimate that the DLS threshold is 80% of car and public transport in total transport modes, and the occupancy rate of other transport modes is kept constant over time. We estimate the minimum mobility demand to be 10,000 p-km across all transport modes^34^. Accordingly, we estimate the national gap of mobility below the threshold based on the average p-km per region by modal share (car and public transport).

#### Water

Normative requirements include in-house or accessible, safe water supply. For Water, the data of people using safely managed drinking water services (% of population) is from the World Bank^51^. The “safe water supply” represents people using safely managed drinking water services. Drinking water from an improved water source is located on the premises, available when needed, and free from fecal and priority contamination, which considers the accessibility, availability, and quality of the household water source.

#### Sanitation

For Sanitation, the data of people using safely managed sanitation services (% of population) is from the World Bank^51^. Then we apply the emissions intensity of the Sanitation sector (CO_2_ Mt per dollar) by using EMERGING MRIO to estimate emission requirements in the Sanitation indicator. The “safely managed sanitation services” represent that population using an improved sanitation facility that is not shared with other households and where excreta is safely disposed of in situ or treated off-site.

#### Electricity

For Electricity, we set a normative DLS threshold as 100 percent of the population has access to electricity in all countries. The data on access to electricity (% of population) is from the World Bank^51^. Then we apply the emissions intensity of the Electricity sector (CO_2_ Mt per dollar) by using EMERGING MRIO to estimate emission requirements.

#### Clothing

Clothing expenditure is shown to be strongly associated with life expectancy and infant mortality, which are highly relevant to poverty^53^. Due to the lack of a unified standard for the threshold requirement of Clothing indicators in previous literatures^14,18^, which only requires these clothes are only adequate for daily activities under local climatic conditions. Therefore, we have set a normative DLS threshold for the Clothing indicator at PPP$500 per capita, calculated based on the total amount required to purchase a set of summer clothing (tops, bottoms, and shoes) and a set of winter clothing (tops, bottoms, and shoes) at US dollar prices. We devise the national gap in clothing based on the annual per capita clothing expenditure calculated by EMERGING MRIO. Based on the embodied emission intensities (CO_2_ Mt per dollar) and final household consumption of related Clothing/footwear sectors by using EMERGING MRIO, we derive the total final emission footprints for the Clothing indicator.

#### Health

We devise the national gap of health based on the annual per capita health expenditure in the Global Health Expenditure Database (GHED) ^54,55^. We set a normative DLS threshold for Health indicators as minimum national health expenditure (PPP$450 per cap), based on Rao et al.^14^

#### Housing

Normative requirements for Housing include (1) no population living in slums from UN-Habitat^56^; and (2) adequate AC installation levels^14,57,58^. The Housing gap is determined according to the data of UN-Habitat on the population currently living in slums^59–61^, and then we combine the per capita floor area threshold to calculate housing needs in emerging economies. The normative DLS floor area threshold is 10 m^2^ of residential floor area per capita^18^.

We assume that the AC of each country is related to the climate conditions of that country. We calculate AC demand by identifying the proportion of the population in specific climate zones in different countries. As for AC availability in different countries, based on the collected national AC data and per capita GDP data in limited economies^62^, we assume the availability of AC follows the Logistic distribution of GDP per capita^63^ and the AC availability in other economies as follows:

1 $${{{{{\rm{Avalibility}}}}}}=\frac{1}{1+{e}^{4.152}\times {e}^{-0.237\times {{{{{\rm{Income}}}}}}}}$$

where income is defined as GDP in PPP per capita (thousand dollars). Note: this part of the emissions requirement finally belongs to the Electricity indicator.

For carbon footprint accounting of DLS indicators, MRIO analysis is used to derive emission intensities for food, clothing, health, water, sanitation, ICT, and education, and we use emission intensity by life cycle assessment (LCA) for mobility, housing, and electricity. The method selection is based on the consistency between the material requirements of different DLS indicators and the sector or product definition in each method.

### Consumption-based carbon footprint accounting

Generally, we use the LCA method for the capital-intensive and product-specific dimensions, including mobility, housing, and electricity in AC appliances. We rely on MRIO analysis for the remaining non-product-specific dimensions, including food, water, sanitation, clothing, health, and education^18^.

#### MRIO analysis

The global MRIO framework accounts balance of monetary flows between economic sectors and regions, which can be written as:

2 $${{{{{\bf{x}}}}}}={\left({{{{{\bf{I}}}}}}-{{{{{\bf{A}}}}}}\right)}^{-1}{{{{{\bf{y}}}}}}$$

where $\,{{{{{\bf{X}}}}}}$ is the sectoral output for all countries; ${({{{{{\bf{I}}}}}}-{{{{{\bf{A}}}}}})}^{-1}$ is the Leontief inverse matrix; and ${{{{{\bf{y}}}}}}$ is the final demand (household, government, and capital) for all economies.

To calculate the consumption-based carbon footprints of DLS indicators (except mobility, housing, and electricity), environmental input-output analyses have been widely used to illustrate the environmental impact caused by economic activities^64–68^. Among this, the sectoral emission intensity for all regions^69^, calculated from the production side, $\,{{{{{\bf{k}}}}}}$:

3 $$\begin{array}{cc}{k}_{i}={V}_{i}/{x}_{i} & {{{{{\rm{for}}}}}}\,i=1\,{{{{{\rm{to}}}}}}\,N\end{array}$$

where $k_{i}$ is the direct CO_2_ emission intensity of sector *i*. ${V}$ is the direct CO_2_ emissions generated by sector *i*,$\,{{{{{\bf{x}}}}}}$ is the total output of sector *i*.

Thus, the CO_2_ emission footprints for the seven DLS indicators can be calculated by:

4 $${{{{{\bf{E}}}}}}={{{{{\bf{k}}}}}}{({{{{{\bf{I}}}}}}-{{{{{\bf{A}}}}}})}^{-1}{{{{{\rm{diag}}}}}}{({{{{{\bf{y}}}}}}_{{{{{\bf{H}}}}}})}$$

where **E** is the CO_2_ emission footprint vector (both direct and indirect); ${{{{{\bf{k}}}}}}$ is the vector of direct CO_2_ emissions intensity by sector.$\,{{{{{\rm{diag}}}}}}{({{{{{\bf{y}}}}}}_{{{{{\bf{H}}}}}})}$ is the diagonalized final demand of household supply for all economies.

#### Life cycle analysis

LCA is a method used to evaluate the environmental impacts of products over the whole life cycle, which is from the acquisition of raw materials, through the production of products, to the disposal of products after use^70^. The following LCA studies have been used for the embodied emission intensity of DLS indicators:

##### Mobility

Average emission intensity for cars: Levon Amatun^71^, 228 g CO_2_-eq/km (4 passengers per car); Average emission intensity for buses: Levon Amatuni^71^, 187 g CO_2_-eq/km (30 passengers per bus); Average emission intensity for motorcycles: Gerson Carranza^72^, 97 g CO_2_-eq/km (1 passenger per motorcycle); Average emission intensity for bicycles: Levon Amatuni^71^, 21 g CO_2_-eq/km (1 passenger per bicycle).

The CO_2_ emission footprint for mobility can be calculated by:

5 $${{E}}=10,000\,*\,\bigg({k}_{{{{{\rm{car}}}}}}\times \left(p\,*\,{R}_{{{{{\rm{car}}}}}}\right)/4+{k}_{{{{{\rm{bus}}}}}}\times \left(p\,*\,{R}_{{{{{\rm{bus}}}}}}\right)/30+{k}_{{{{{\rm{motor}}}}}}\\ \times \left(p\,*\,{R}_{{{{{\rm{motor}}}}}}\right)+{k}_{{{{{\rm{bicy}}}}}}\times \left(p\,*\,{R}_{{{{{\rm{bicy}}}}}}\right)\bigg)$$

where *E* is the CO_2_ emission footprint vector (both direct and indirect); ${{{{{\boldsymbol{p}}}}}}$ is the total population of each country; ${{{k}}}_{{{{{{\rm{car}}}}}}}{{,}}\,{{{k}}}_{{{{{{\rm{bus}}}}}}}$,${{{k}}}_{{{{{{\rm{motor}}}}}}}$, and ${{k}}_{{{{{{\rm{bicy}}}}}}}$ are the average emission intensity for cars, buses, motorcycles, and bicycles; ${{R}}_{{{{{{\rm{car}}}}}}}\,{{R}}_{{{{{{\rm{bus}}}}}}}$, ${{R}}_{{{{{{\rm{motor}}}}}}}$, and ${{R}}_{{{{{{\rm{bicy}}}}}}}$ are the availability rate of transportation modes for the national population for cars, buses, motorcycles, and bicycles; the minimum mobility demand is 10,000 p-km (See in DLS indicator processing part).

##### Housing

Average emission intensity for residential buildings: Ž. Tomková^73^, 40 kg CO_2_-eq/m^2^.

The CO_2_ emission footprint for housing can be calculated by:

6 $${{E}}=10\,*\,({{{k}}}_{{{{{{\rm{housing}}}}}}}\times \left({{p}}\,*\,{{{R}}}_{{{{{{\rm{slum}}}}}}}\right))$$

where *E* is the CO_2_ emission footprint vector (both direct and indirect); ${{{{{\boldsymbol{p}}}}}}$ is the total population of each country;$\,{{k}}_{{{{{{\rm{housing}}}}}}}$ is the emission intensity for residential buildings; ${{R}}_{{{{{{\rm{slum}}}}}}}$ is the population share who is living in slums; the normative DLS threshold of the floor area is 10 m^2^ living space of residential buildings per capita (see in DLS indicator processing part).

##### Electricity

For the CO_2_ emissions resulting from AC demand, we assume that each family’s (including three persons on average) AC maintains the temperature at 25 °C. And the average emission intensity for AC: Ross and Cheah^74^, 3656 kg CO_2_-eq per AC.

And the CO_2_ emission footprint for AC demand can be calculated by:

7 $${{E}}={k}_{{{{{\rm{AC}}}}}}\times \left(p\,*\,{R}_{{{{{\rm{zone}}}}}}\right)$$

where *E* is the CO_2_ emission footprint vector (both direct and indirect); ${{{{{\boldsymbol{p}}}}}}$ is the total population of each country; $\,{{k}}_{{{{{{\rm{AC}}}}}}}$ is the emission intensity for AC demand; ${{R}}_{{{{{{\rm{zone}}}}}}}$ is the population share who is living in slums (see in DLS indicator processing part).

The lack of data samples from LCA literature poses a challenge in estimating CO_2_ emission increments accurately. Assuming that the emission intensity of all countries is the same for all indicators, such as mobility, AC, and housing, can introduce a certain level of error and uncertainty into our estimation. To address this issue, we have adjusted the CO_2_ emission intensity of LCA by using the normalized corresponding MRIO sectoral emission intensity of EMERGING MRIO in different countries to reflect regional differences in the carbon emission intensity of corresponding DLS indicators. For instance, for the Housing indicator, we have used the sectoral emission intensity of the Construction sector; for AC demand, we have used the sectoral emission intensity of the Electricity sector; and for the Mobility indicator, we have used the average sectoral emission intensity of Sea transport, Air transport, Other modes of transport, and Local transport sectors.

### National emission reduction commitments calculation

The original national emission mitigation policy information of 121 emerging economies is from the Net Zero Tracker^22^, which mainly includes the interim target and end target. In this study, as we focus on the impact of CO_2_ emissions increments generated for the rapid achievement of DLS on national carbon emissions reductions, we use interim targets for CO_2_ emission reduction commitment standards (mainly 2030 and 2025). The parameters provided by the carbon emission reduction policies from the Net Zero Tracker include target base year ${{{{{\rm{y}}}}}}_{{{{{\rm{base}}}}}}$, target emission reduction percentage ${p}_{{{{{\rm{emission}}}}}}$ (or target carbon emission intensity emission reduction percentage ${p}_{{{{{\rm{intenisty}}}}}}$). Based on the total CO_2_ emissions ${E}_{{{{{\rm{ybase}}}}}}$ (or carbon emission intensity ${I}_{{{{{\rm{ybase}}}}}}$) of the target base year from the IEA and the national GDP in 2019 (${{{{{\rm{GDP}}}}}}_{{{{{\rm{2019}}}}}}$) from the World Bank, we finally estimate the total carbon emission reduction commitments of emerging economies ${{{{{\rm{ERC}}}}}}_{{{{{\rm{target}}}}}}$ in Eqs. (8) or (9):

8 $${{{{{\rm{ERC}}}}}}_{{{{{\rm{target}}}}}}={p}_{{{{{\rm{emission}}}}}}\times {E}_{{{{{\rm{ybase}}}}}}$$

9 $${{{{{\rm{ERC}}}}}}_{{{{{\rm{target}}}}}}={p}_{{{{{\rm{intensity}}}}}}\times {I}_{{{{{\rm{ybase}}}}}}\times {{{{{\rm{GDP}}}}}}_{{{{{\rm{2019}}}}}}$$

### LS achievement scenarios

We have designed counterfactual LS achievement scenarios, which only focuses on change in household consumption and does not attempt to explain the impact of other contributors (e.g., dynamic implementation of DLS, changes in national production structure, and the variations in carbon emission intensity due to advancements in green production technology) on achieving DLS or the dynamic process of DLS achievement in reality, which follows Bruckner et al. ^13^. In order to compare the additional emissions for LS in different development stages, we have created five different LS scenarios: “USA”: living standard of the USA; “EU”: average living standard of EU27 + UK; “CHN”: living standard of China; “IND”: living standard of India; “DLS”: achieving decent living.

### Uncertainty analysis

Monte Carlo simulation (MCS) is used to analyze the uncertainty of the DLS CO_2_ emission increments results and is one of the most popular methods for studying of parameter uncertainty^75–78^. The essence of MCS is to randomly repeat samples from several probability distributions of input variables to establish the distribution of output variables^79^. According to the provisions in the existing literature, they usually obtain the distribution of general input parameters from the literature or by assuming a Gaussian distribution (Uniform distribution) with a variation range^75,80,81^. According to the uncertainty types determined by the Intergovernmental Panel on Climate Change (IPCC) ^18,82^, we mainly quantified two types of uncertainty: the value uncertainty associated with data input and the model’s structural uncertainty related to carbon footprint calculation.

Regarding the input data values, we conducted the sensitivity analysis on the data of DLS indicators and threshold parameters. 7 DLS indicators (Food, Electricity, Sanitation, Health, Education, Water, and ICT) are obtained from international statistical databases at the national level, such as the World Bank, FAOSTAT, and UN (Supplementary Table 1), and have been widely used for global economic analysis^15,64^. Therefore, we consider their feasibility to be high and have set the small uncertainty range at ±5%. The Housing indicator has two aspects. The AC availability (Housing2) is calculated based on the assumption that it follows the Logistic distribution of GDP per capita, which introduces inherent uncertainty, resulting in an uncertainty range of ±15%. The proportion of the population living in slums (Housing1) has a lower uncertainty range of ±5%, sourced from the UN-Habitat. The Mobility indicator’s uncertainty range is set at ±10%, as the ITF Transport Statistics only provide the shares of transportation modes for 7 country groups (Supplementary Data 3), which we scaled down to 245 economies worldwide, introducing some degree of error. Due to the lack of data, the Clothing indicator, the annual per capita clothing expenditure, is calculated by using the final household consumption of related Clothing/footwear sectors in EMERGING MRIO. Treating the final consumption of macroeconomic sectors as household expenditure of clothing will introduce some level of uncertainty, especially for emerging economies, with an uncertainty range of ±15% (Supplementary Table 5).

The main uncertainty in our study lies in the EMERGING MRIO and the LCA method. The EMERGING MRIO uses multiple data sources and various economic assumptions to create a global economic model, and its uncertainty range cannot be accurately quantified at present^19^. Therefore, we have set the uncertainty range of emission intensity obtained through the EMERGING MRIO and LCA methods at ±12%, in accordance with the IPCC AR6 WGIII report^1^.

A total number of 1000 MCSs are executed for 11 input parameters:10 DLS indicators (within the range ±5 to 15%) and emission intensity (within the range ±12%), which follow a Gaussian distribution, to obtain one output parameter “DLS”. The uncertainty of DLS CO_2_ emission increments is finally explained by combining the upper limit of uncertainty (95th percentile) and the lower limit of uncertainty (5th percentile) with acceptable values. We have compiled the uncertainty results of the above parameters and the overall results into Supplementary Table 5.

### Supplementary information

Supplementary Information

Description of Additional Supplementary Files

Supplementary Data 1

Supplementary Data 2

Supplementary Data 3

Supplementary Data 4

Supplementary Data 5

## Data Availability

The multi-regional input-output table (EMERGING) for 2019 can be downloaded from the CEADs website (https://ceads.net/user/index.php?id=1274&lang=en) free of charge. CO_2_ emissions from fossil fuel combustion and energy consumption at the sectoral level in each region are available from the IEA database and CEADs emission inventory data. Data supporting the findings of this study have been deposited in figshare (10.6084/m9.figshare.24167874).

## Code availability

The code developed in Matlab and R to process and analyze the primary data collected in this study will be reviewed and made available upon request.
