# Supplementary material for: Achieving decent living standards in emerging economies challenges national mitigation goals for CO2 emissions
Source: Nat Commun. 2023 Oct 10;14:6342. doi: 10.1038/s41467-023-42079-8 (PMC10564770; doi:10.1038/s41467-023-42079-8)
Supplement: Supplementary file 1 — Supplementary Information [file 41467_2023_42079_MOESM1_ESM.pdf]

Supplemental Information

Achieving a decent living in emerging economies challenges national reductions goals

Jingwen Huo, Jing Meng, Heran Zheng, Priti Parikh, Dabo Guan

Supplementary Figures

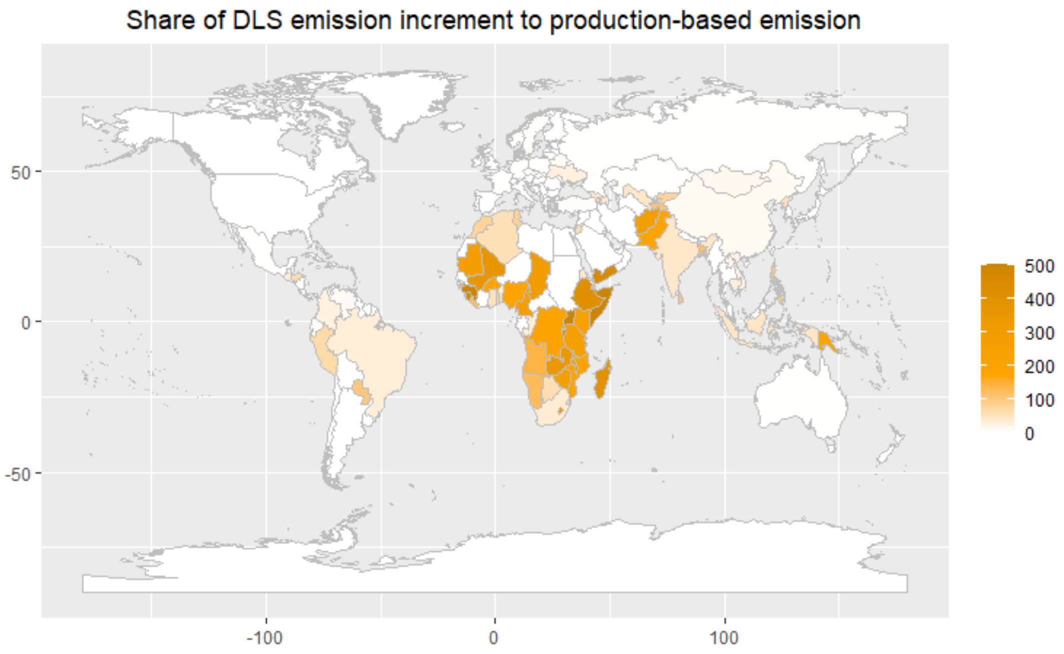

Supplementary Fig. 1. The increment shares of CO_2 emission for achieving DLS compared with the national production-based emissions in 2019. The base map is from the mapdata package (TM World Borders Dataset 0.3) in R (https://search.r-project.org/CRAN/refmans/prevR/html/TMWorldBorders.html).

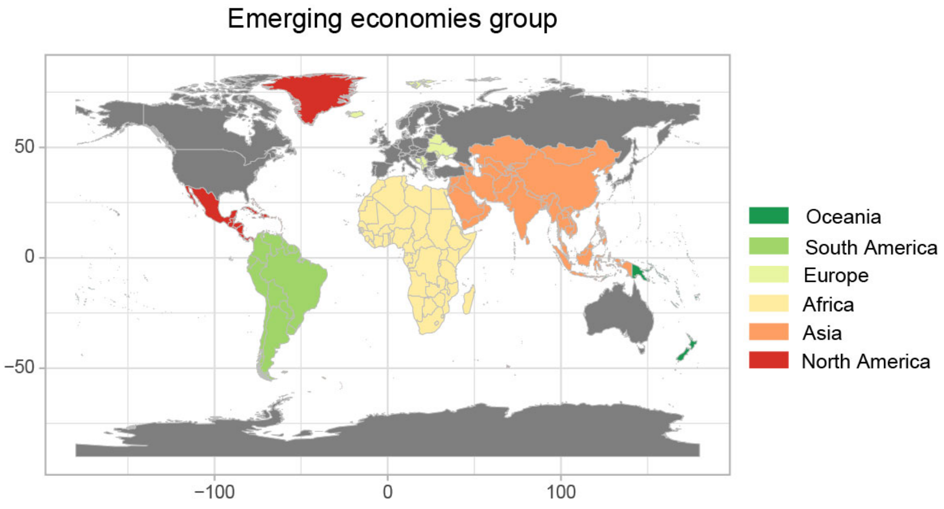

Supplementary Fig. 2. Regional distribution of global emerging economies. The base map is from the mapdata package (TM World Borders Dataset 0.3) in R (https://search.r-project.org/CRAN/refmans/prevR/html/TMWorldBorders.html).

Supplementary Tables

Supplementary Table 1. Raw data sources of 10 DLS indicators. Due to data availability, we use UN-Habitat data on the population currently living in slums in 2018 and assume that the share of the population currently living in slums in 2019 is the same as in 2018.

Indicator	Unit	Time	Data source
**Food**	kcal/capita/day	2019	FAOSTAT: https://www.fao.org/faostat/en/#data/FBS
**Housing**	share %	2018*	UN-Habitat: https://data.unhabitat.org/datasets/proportion-of-urban-population-living-in-slum-households-by-country-or-area-1990-2018-percent/explore
**Electricity**	share %	2019	World Bank: https://data.worldbank.org/
**Clothing**	$ PPP/cap	2019	EMERGING MRIO
**Water**	share %	2019	World Bank: https://data.worldbank.org/
**Sanitation**	share %	2019	World Bank: https://data.worldbank.org/
**Education**	share %	2019	World Bank: https://data.worldbank.org/
**Health**	$ PPP/cap	2019	Global Health Expenditure Database: https://apps.who.int/nha/database/Select/Indicators/en
**Mobility**	p-km/cap (%)	2019	ITF Transport Statistics: https://stats.oecd.org/BrandedView.aspx?oecd_bv_id=trsprt-data-en&doi=4785726e-en
**ICT**	share %	2019	UN Data: https://data.un.org/

Supplementary Table 2. DLS requirements in kcal per cap per day based on the population structure.

Age group	Male	Female
0-4	1300	1300
5-9	2032	1936
10-14	2629	2342
15-19	3155	2462
20-64	2700	3000
>64	2100	2500

Supplementary Table 3. Country list of 50 emerging economies, whose historical energy-related CO_2 emission data is from CEADs emission inventory data.

**Country**	**Region**
Cambodia	Asia
Laos	Asia
Myanmar	Asia
India	Asia
Indonesia	Asia
Jordan	Asia
Mongolia	Asia
Thailand	Asia
turkey	Asia
Djibouti	Africa
Ethiopia	Africa
Tanzania	Africa
Uganda	Africa
Ghana	Africa
Kenya	Africa
South Africa	Africa
Bolivia	South America
Guatemala	North America
Jamaica	North America
Ecuador	South America
Paraguay	South America
Colombia	South America

Peru	South America
Brazil	South America
Chile	South America
Argentina	South America
Uruguay	South America
Moldova	Europe
Russia	Europe
Estonia	Europe
Egypt	Africa
Cuba	North America
Morocco	Africa
Burundi	Africa
Rwanda	Africa
Madagascar	Africa
Niger	Africa
Panama	North America
Micronesia	Oceania
Liberia	Africa
Togo	Africa
Tunisia	Africa
Nigeria	Africa
Mauritius	Africa
Sri Lanka	Asia
Algeria	Africa

Israel	Asia
Philippines	Asia
Saudi Arabia	Asia
Nicaragua	North America

Supplementary Table 4. The CO_2 emissions requirement if developed countries only meet the minimum standards of DLS, compared with that in 2019. D_2019 means the CO_2 emissions for LS of all the developed economies in 2019; DLS means the CO_2 emissions for meeting the minimum DLS standards of all the developed economies.

CO_2 Mt	**D_2019**	**D_DLS**
Food	493.0	396.1
ICT	56.3	92.6
Education	71.3	73.7
Mobility	840.2	819.5
Water	58.2	4.3
Sanitation	13.9	15.3
Electricity	815.9	823.6
Housing	2.6	2.7
Clothing	159.8	269.5
Health	431.3	30.5
Total	2942.4	2527.8

*D_2019 means the CO_2 emissions for LS of all the developed economies in 2019;
**DLS means the CO_2 emissions for meeting the minimum DLS standards of all the developed economies;

Supplementary Table 5. Uncertainty analysis of the required CO_2 emissions for LS in 2019 and for achieving DLS (10 DLS indicators). a UR: the uncertainty range of each DLS indicator and emission intensity; b 2019: the required CO2 emissions for LS in 2019; c DLS: the required CO2 emission for achieving DLS; d CI_2019: 95% confidence interval for the CO2 emission requirements for LS in 2019; e CI_DLS: 95% confidence interval for the CO2 emission requirements for achieving DLS; f Housing1: The proportion of the population currently living in slums; g Housing2: national AC availability

		**UR**[a]	**2019**[b]	**CI_2019**[c]	**DLS**[d]	**CI_DLS**[e]
Food		(-5%, 5%)	896.1	(859.8, 936.1)	898.9	(862.7, 938.6)
ICT		(-5%, 5%)	244.1	(237.7, 250.2)	595.3	(579.6, 612.0)
Education		(-5%, 5%)	27.3	(26.5, 28.2)	35.9	(34.8, 37.2)
Mobility		(-10%, 10%)	2681.5	(2523.5,2839.8)	2990.1	(2794.3, 3192.0)
Water		(-5%, 5%)	126.7	(123.3, 130.2)	127.1	(123.6, 130.5)
Sanitation		(-5%, 5%)	5.9	(5.5, 6.3)	9.3	(8.8, 9.9)
Electricity		(-5%, 5%)	1946.6	(1771.4, 2109.1)	9454.6	(8738.0, 10165.2)
Housing	Housing1[f]	(-5%, 5%)	21.8	(20.2, 23.5)	34.9	(32.3, 37.7)
	Housing2[g]	(-15%, 15%)				
Clothing		(-15%, 15%)	161.7	(152.9, 170.2)	573.1	(534.1, 601.7)
Health		(-5%, 5%)	36.8	(35.8, 37.8)	41.8	(40.6, 42.9)
Emission intensity		(-12%, 12%)				
Total			6148.6	(5907.8, 6386.2)	14761.0	(13983.4, 15544.0)
