# Supplementary material for: Achieving decent living standards in emerging economies challenges national mitigation goals for CO2 emissions
Source: Nat Commun. 2023 Oct 10;14:6342. doi: 10.1038/s41467-023-42079-8 (PMC10564770; doi:10.1038/s41467-023-42079-8)
Supplement: Supplementary file 2 — Description of Additional Supplementary Files [file 41467_2023_42079_MOESM2_ESM.pdf]

Description of Additional Supplementary Files

File Name: Supplementary Data 1
Description: 245 countries and classification of EMERGING

File Name: Supplementary Data 2
Description: Sector classification and mapping for 135 sectors of EMERGING

File Name: Supplementary Data 3
Description: 245 economies and classification of 7 country groups in ITF Transport Statistics

File Name: Supplementary Data 4
Description: The value of 10 DLS indicators for all the economies

File Name: Supplementary Data 5
Description: Detail results of national CO_2 emission reduction commitments and emission increments of DLS for 121 emerging economies
